# Why Is Preeclampsia still an Important Cause of Maternal Mortality Worldwide?

**DOI:** 10.1055/s-0040-1714132

**Published:** 2020-09-29

**Authors:** Thaís Alquezar Facca, Eduardo Augusto Brosco Famá, Gianna Mastroianni-Kirsztajn, Nelson Sass

**Affiliations:** 1 Division of Nephrology, Escola Paulista de Medicina, Universidade Federal de São Paulo, São Paulo, SP, Brazil; 2 Department of Obstetrics, Escola Paulista de Medicina, Universidade Federal de São Paulo, São Paulo, SP, Brazil

According to the World Health Organization, ∼ 810 women died per day from preventable causes related to pregnancy in 2017. Most of these deaths occurred in low/lower middle-income countries and in low-resource settings. The maternal mortality rate worldwide is still high, even though it dropped by 38% between 2000 and 2017. 1

As highlighted in the latest report of the American College of Obstetricians and Gynecologists, preeclampsia is responsible for 50,000 to 60,000 deaths/year and ∼ 50 to 100 maternal near-misses for every preeclampsia-related death. Despite of the advances in the understanding of preeclampsia, the clinical practice does not seem to be progressing at the same rate. 2

Hypertensive disorders, including preeclampsia, are very common complications of pregnancy, with an incidence of 5 to 10%. 3 However, this incidence might be underestimated due to underreporting. Health professionals at all levels of medical care need to know the clinical and laboratory manifestations to diagnose this disease. That is why protocols are required to guide physicians to know how to deal with hypertension in pregnancy and achieve better maternal and perinatal outcomes, especially in the developing countries. 4

In the last few years, there has been a decrease in the rates of complications related to preeclampsia in the developed countries, but this is not being observed in the developing ones, probably due to the lack of hospital resources and failure of medical and prenatal care. 5 Besides that, the incidence of preeclampsia has increased by 25% in the United States in the past 20 years for unknown reasons. 2

Stroke, HELLP syndrome (Hemolysis, Elevated Liver Enzymes, Low Platelets), eclampsia, hemorrhage, and cardiopulmonary and renal complications are the main causes of maternal mortality associated with preeclampsia. Clinical warning signs, high blood pressure, and laboratory abnormalities are important variables that need to be consistently observed to improve the immediate treatment. 6 Hopefully, due to the widespread use of magnesium sulfate and the improved prenatal care, the rate of eclampsia has decreased. 7

A difficulty in the medical care of women with hypertension is that, when they have signs or symptoms of severe preeclampsia, the disease may progress rapidly to clinical worsening or death, and this may occur during pregnancy, labor, or even in the postpartum period. 8 For this reason, an online calculator was recently developed to predict severe maternal outcomes, called full preeclampsia integrated estimate of risk (PIERS) calculator. This tool provides the percentage of risk of having adverse maternal outcomes within 48 hours up to a week after assessment by analyzing gestational age, presence of dyspnea or chest pain, O _2_ saturation, levels of serum creatinine, and liver transaminases. 9

Currently, only the use of calcium and low-dose aspirin are considered effective for preventing preeclampsia. Therefore, the adequate prenatal care is an important preventive measure allowing clinicians to prescribe these medications at the appropriate time and detect risk factors such as previous personal or family history of preeclampsia, chronic hypertension, kidney disease, diabetes, autoimmune disorders of connective tissue, thrombophilia, black ethnicity, obesity, and primigravida. 8

In conclusion, preeclampsia is still an important cause of maternal mortality, acute and long-term complications worldwide, and this is an alert for physicians. Aiming to improve maternal and perinatal outcomes, protocols are essential in the health services to guide professionals for the care of pregnant women with arterial hypertension. This measure may optimize early detection, treatment and prevention of the disease, especially in developing countries.
